# Expected Value of Sample Information Calculations for Risk Prediction Model Validation

**DOI:** 10.1177/0272989X251314010

**Published:** 2025-02-18

**Authors:** Mohsen Sadatsafavi, Andrew J. Vickers, Tae Yoon Lee, Paul Gustafson, Laure Wynants

**Affiliations:** Respiratory Evaluation Sciences Program, Collaboration for Outcomes Research and Evaluation, Faculty of Pharmaceutical Sciences, The University of British Columbia, Vancouver, BC, Canada; Department of Epidemiology and Biostatistics, Memorial Sloan, Kettering Cancer Center, New York, NY, USA; Respiratory Evaluation Sciences Program, Collaboration for Outcomes Research and Evaluation, Faculty of Pharmaceutical Sciences, The University of British Columbia, Vancouver, BC, Canada; Department of Statistics, The University of British Columbia, Vancouver, BC, Canada; Department of Epidemiology, CAPHRI Care and Public Health, Research Institute, Maastricht University, Maastricht, The Netherlands; Department of Development and Regeneration, KU Leuven, Leuven, Belgium

**Keywords:** risk prediction, value of information, uncertainty, Bayesian statistics

## Abstract

**Background:**

The purpose of external validation of a risk prediction model is to evaluate its performance before recommending it for use in a new population. Sample size calculations for such validation studies are currently based on classical inferential statistics around metrics of discrimination, calibration, and net benefit (NB). For NB as a measure of clinical utility, the relevance of inferential statistics is doubtful. Value-of-information methodology enables quantifying the value of collecting validation data in terms of expected gain in clinical utility.

**Methods:**

We define the validation expected value of sample information (EVSI) as the expected gain in NB by procuring a validation sample of a given size. We propose 3 algorithms for EVSI computation and compare their face validity and computation time in simulation studies. In a case study, we use the non-US subset of a clinical trial to create a risk prediction model for short-term mortality after myocardial infarction and calculate validation EVSI at a range of sample sizes for the US population.

**Results:**

Computation methods generated similar EVSI values in simulation studies, although they differed in numerical accuracy and computation times. At 2% risk threshold, procuring 1,000 observations for external validation, had an EVSI of 0.00101 in true-positive units or 0.04938 in false-positive units. Scaled by heart attack incidence in the United States, the population EVSI was 806 in true positives gained, or 39,500 in false positives averted, annually. Validation studies with >4,000 observations had diminishing returns, as the EVSIs were approaching their maximum possible value.

**Conclusion:**

Value-of-information methodology quantifies the return on investment from conducting an external validation study and can provide a value-based perspective when designing such studies.

**Highlights:**

- In external validation studies of risk prediction models, the finite size of the validation sample leads to uncertain conclusions about the performance of the model. This uncertainty has hitherto been approached from a classical inferential perspective (e.g., confidence interval around the c-statistic).
- Correspondingly, sample size calculations for validation studies have been based on classical inferential statistics. For measures of clinical utility such as net benefit, the relevance of this approach is doubtful.
- This article defines the expected value of sample information (EVSI) for model validation and suggests algorithms for its computation. Validation EVSI quantifies the return on investment from conducting a validation study.
- Value-based approaches rooted in decision theory can complement contemporary study design and sample size calculation methods in predictive analytics.

Risk prediction models that quantify the probability of clinical events are a key aspect of individualized medicine. Once developed, these models are often used in diverse populations. Before being trusted for clinical use in a new population, a prediction model needs to undergo validation in a representative sample from that population.^1^ For example, the original Framingham risk scores for cardiovascular disease risk are developed using data from the Framingham County in the United States but are externally validated for use in many different populations.^2^

During a validation study, the performance of the prediction model is assessed in terms of average prediction error, discrimination (e.g., c-statistic), calibration (e.g., calibration intercept and slope), and net benefit (NB).^3^ Among such metrics, NB is a decision-theoretic one, as it enables direct assessment of the clinical utility of a model (i.e., if a model’s expected NB (ENB) is higher than that of alternatives, it is expected to confer clinical utility). Due to such decision-theoretic underpinnings, and despite its relatively new arrival in the field of predictive analytics, NB has gained significant momentum and has become a standard component of modern external validation studies.^4^

When interpreting the results of a validation study, the finite size of the validation sample means that the assessment of model performance is accompanied by uncertainty. Uncertainty in conventional metrics of model performance is communicated using classical inferential methods (e.g., 95% confidence interval [CI] around the c-statistic or calibration slope). Similarly, sample size considerations when planning a validation study are based on inferential statistics.^5,6^ However, the relevance of this approach to uncertainty around NB as a decision-theoretic metric is doubtful, as arbitrary significance levels or prespecified confidence bands do not necessarily translate to better decisions.^7–9^

Decision theory provides an alternative view to the consequences of uncertainty by relating it to the outcome of decisions. A decision maker should adopt the strategy with the highest expected utility, which, if implementation costs are comparable, is the one with the highest ENB. While uncertainty around NB should not affect the adoption decision, it is associated with utility loss as it hinders our ability to identify the decision with the highest true NB. The extent of uncertainty should thus inform the research decision: whether the expected utility loss is large enough to necessitate collecting further evidence. This approach toward uncertainty quantification is referred to as value-of-information (VoI) analysis.^10–12^

VoI methods are widely accepted in health policy making.^11,13^ However, their relevance to clinical decision making and risk prediction has only recently been highlighted.^14^ In particular, the expected value of perfect information (EVPI), the expected gain in NB by completely eliminating uncertainty, has been applied to both the development and validation phases of risk prediction models.^14,15^ However, to the best of our knowledge, the expected value of sample information (EVSI), the expected gain in NB by conducting a study of a given sample size, has not been defined and applied to clinical prediction models.

The aim of the present work is to define EVSI for the external validation studies of risk prediction models and propose algorithms for its computation. The rest of this article is structured as follows. After outlining the context, we define EVSI for a future validation study aimed at evaluating the clinical utility of a model in a new population. We propose different algorithms for EVSI computations, elaborate on their use case, and compare their performance in simulation studies. A case study puts the developments in context. We conclude by proposing areas of further inquiry.

## Methods

### Context

We focus on validating a previously developed risk prediction model in a new target population. The developments are presented for prediction models for binary responses but are generally applicable to any context where NB can be assessed (e.g., survival outcomes,^16^ models for treatment benefit^17^). A risk prediction model is advertised as a function that maps patient characteristics to an estimate of event risk. We have some current information about the performance of this model in the target population (for example, from expert opinion or from a pilot study) but are uncertain if using the model in this population is net beneficial. We are planning to obtain a random sample D containing N observations from this population to reduce such uncertainty. This sample is expected to improve our knowledge about model performance, which in turn will increase our chance of making the correct decision whether to use the model in this population. As such, procuring such a sample might be associated with NB gain, and we are interested in evaluating the expected gain in NB as a function of N . We acknowledge the relevance of other sources of uncertainty (e.g., missing data, the plausibility that the sample is truly representative of the target population) but focus on sampling uncertainty—that is, our uncertainty in true NBs because of the finite size of the validation sample.

### NB Calculations for Risk Prediction Models

Details of NB calculations for risk prediction models are explained in its original article and in many tutorials.^4,18,19^ In brief, to turn a continuous predicted risk to a binary classification to inform a treatment decision, a decision maker needs to specify a risk threshold z on predicted risks such that individuals classified as high risk are selected for the treatment (e.g., a 7.5%–10% threshold on the Framingham Risk Score is often used to initiate treatment with statins to reduce cardiovascular disease risk^20^). Compared with treating no one (with a default utility of 0), the utility of this classification can be expressed as $p_{\mathrm{tp}}-\omega p_{\mathrm{fp}}$ , where $p_{\mathrm{tp}}$ and $p_{\mathrm{fp}}$ are the probabilities of a true positive and a false positive classification, respectively, at this threshold, and ω≥0 represents the utility tradeoff (exchange rate) between a true-positive and a false-positive classification. Vickers and Elkin showed that the value of ω can be deduced from the risk threshold itself^18^: if a decision maker is willing to treat those with risk >z and not treat those with risk <z , it means that individuals with a risk precisely equal to z will be ambivalent about the treatment decision. This ambivalence indicates that in a population whose average outcome risk is z , the expected utility of treating everyone and treating no one is the same to the decision maker. Because in this population, treating everyone corresponds to $p_{\mathrm{tp}}=z$ and $p_{\mathrm{fp}}=1-z$ , we have ω=z/(1−z) . For example, a 10% threshold on cardiovascular risk for initiating statin therapy means the benefit of treating 1 individual who will experience a cardiovascular event is considered equal to the harm of treating 9 individuals who will not experience such an event without treatment. This exchange rate enables calculating NB in either net true-positive or net false-positive units for a given threshold. In practice, the NB is calculated across a plausible range of thresholds.

It is often more intuitive to express $p_{\mathrm{tp}}$ and $p_{\mathrm{fp}}$ in terms of outcome prevalence and the model’s true sensitivity and specificity in the target population.^21^ Let θ be the triplet of outcome prevalence ( $\theta_{p}$ , a quantity that is independent of model performance), sensitivity ( $\theta_{\mathrm{se}}$ ), and specificity ( $\theta_{\mathrm{sp}}$ ). We have $p_{\mathrm{tp}}=\theta_{p}\theta_{\mathrm{se}}$ and $p_{\mathrm{fp}}=\left(1-\theta_{p}\right)\left(1-\theta_{\mathrm{sp}}\right)$ (for brevity, we drop the notation that indicates sensitivity and specificity are functions of z ).

Any given model should be compared with at least 2 “default” strategies of treating no one and treating all. Treating no one has NB = 0 by definition, and NB calculations for treating all follow the same logic as above but by considering all individuals as positive. Thus, we have 3 competing strategies: do not treat anyone, use the model to treat those with predicted risk ≥z , and treat all. We index them by 0, 1, and 2, respectively. This results in the following equation for NBs:

(1) $$\begin{array}{c} \mathrm{NB}\left(i,\theta \right)=\\ \left\{ \begin{array}{ccc} 0 & i=0 & \left(\mathrm{treat}\;\mathrm{no}\;\mathrm{one}\right)\\ \theta_{p}\theta_{\mathrm{se}}-\left(1-\theta_{p}\right)\left(1-\theta_{\mathrm{sp}}\right)\frac{z}{1-z} & i=1 & \left(\mathrm{use}\;\mathrm{model}\;\mathrm{to}\;\mathrm{decide}\right)\\ \theta_{p}-\left(1-\theta_{p}\right)\frac{z}{1-z} & i=2 & \left(\mathrm{treat}\;\mathrm{all}\right) \end{array}\right., \end{array}$$

For time-to-event outcomes, θ s and thus NBs are time dependent and should be defined at a time-horizon of interest.^16^ If there are alternative strategies, they can also be considered (the above indexing of NB() can accommodate other strategies), but this is omitted here for ease of exposition.

### VoI Analysis

VoI analysis is a Bayesian approach and requires explicit specification of current information on the performance of the model in the target population. By default, we assume this information is generated from a preliminary validation exercise based on a random sample d of size n . However, such information can be generated in other ways. For example, one can extrapolate the performance of a model from its development sample or its performance in previous validation studies^22^ and accommodate any added uncertainty about the compatibility of the populations by quantitatively discounting external information.^23^ Formal expert elicitation approaches can also be used to construct distributions around model performance metrics.^24^ In such instances, d can be an abstract entity representing, for example, the expert-elicited information.

### The Expected Value of Current Information

Our current information about θ based on the available evidence is expressed as P(θ|d) . According to Bayes’s theorem, P(θ|d)∝P(θ)P(d|θ) , which indicates that this information is influenced by any prior information we might have had, as well as by P(d|θ) , that is, the likelihood function, the support the observed data provide for a given value of θ . With such current information, the decision maker should pick the strategy with the highest ENB given the current information:

(2) $$\mathrm{EN}B_{\mathrm{current}}=\underset{i\in \{0,1,2\}}{\max}E_{\theta |d}\mathrm{NB}\left(i,\theta \right),$$

where the expectation is with respect to P(θ|d) .

### EVPI

Details of the reasoning and algorithms behind calculating EVPI are presented previously.^15^ In brief, if we could know the true values of θ , we would pick the strategy with the highest true NB. We indeed do not have access to the ground truth but have partial information in terms of P(θ|d) . As such, we can model the consequence of knowing the truth (having perfect information) and take its expectation with respect to this distribution:

(3) $$\mathrm{EN}B_{\mathrm{truth}}=E_{\theta |d}\left[\underset{i\in \{0,1,2\}}{\max}\mathrm{NB}\left(i,\theta \right)\right].$$

The EVPI is then the difference in ENB of having perfect information versus current information:

(4) $$\mathrm{EVPI}=\mathrm{EN}B_{\mathrm{truth}}-\mathrm{EN}B_{\mathrm{current}}.$$

The EVPI is a scalar quantity and quantifies the expected loss in NB due to uncertainty, or alternatively, the expected gain in NB by completely resolving uncertainty (i.e., conducting a validation study of infinite size). We have proposed a generic algorithm based on bootstrapping to estimate this expectation as well as an asymptotic approach based on the central limit theorem and 2-dimensional unit normal loss integral.^15^

### EVSI

The reasoning behind the EVSI calculation is similar to that of EVPI, with the modification that instead of knowing the truth, we will have more (but not perfect) information about θ , with additional information coming from obtaining a future sample D . With this added information we will have the opportunity to revise our current decision. We will update our knowledge from P(θ|d) to P(θ|d,D) and will select the strategy that has the highest ENB.

The NB of this approach once future data are obtained is $\underset{i}{\max}E_{\theta |D,d}\mathrm{NB}\left(i,\theta \right)$ . We do not know what data we will observe in the future, but we can treat D as a random variable and take the expectation of this line of reasoning with regard to its predictive distribution (the distribution for future observations given current data and the model):

(5) $$\mathrm{EN}B_{\mathrm{sample}}=E_{D|d}\left[\underset{i\in \{0,1,2\}}{\max}E_{\theta |d,D}\mathrm{NB}\left(i,\theta \right)\right].$$

The EVSI is the difference between this ENB and ENB under current information:

(6) $$\mathrm{EVSI}=\mathrm{EN}B_{\mathrm{sample}}-\mathrm{EN}B_{\mathrm{current}}.$$

The EVSI is a nonnegative scalar quantity in the same NB units as EVPI. It quantifies the expected gain in NB by procuring a validation sample of a given size. The higher the EVSI, the higher the expected gain from the planned validation study. EVPI, quantifying the ENB gain by completely resolving uncertainty, puts an upper limit on EVSI.

## EVSI Computation Algorithms

The challenge for EVSI calculation is the 2 nested expectations in ${\mathrm{ENB}}_{\mathrm{sample}}$ . The inner expectation represents updated estimates of NBs after obtaining future data, and the outer expectation is with respect to the predictive distribution of future data. Below we detail 3 computation algorithms. Exemplary R implementations of the algorithms are provided in the Supplementary Material Section 1.

### A Bootstrap-Based Algorithm

If the current evidence d is in terms of a random sample of size n from the target population (e.g., from a pilot study), EVSI computation can be performed via a 2-level resampling algorithm. This approach has been previously applied to EVSI computation for data-driven economic evaluations^25^ and can be seen as a direct extension of the bootstrap method for EVPI computation for external validation studies.^15^ The logic behind this approach is provided in detail previously.^25^ In a nutshell, let $D^{*}$ be the target population from which d and D are sampled. We take $D^{*}$ as a random entity and apply Bayes’s rule: $P\left(D^{*}|d\right)\propto P\left(D^{*}\right)P\left(d|D^{*}\right)$ . Rubin showed that by using the improper prior $P\left(D^{*}\right)\sim \mathrm{Dirichlet}\left(0,0,\ldots ,0\right)$ placed on all possible observations in $D^{*}$ , the posterior distribution $P\left(D^{*}|d\right)$ will be a discrete distribution that puts random weights Dirichlet(1,1,…,1) on each observation in d . Since any parameter of interest θ is a function of $D^{*}$ , posterior draws of weights from the Dirichlet(1,1,…,1) distribution produce draws from the posterior distribution of P(θ|d) .^26^ Further, once the generating population is at hand, the data of the future study ( D ) can be drawn by sampling from this population. The resulting 2-level resampling algorithm is explained in Table 1.

**Table 1:** Bootstrap-Based Computation of EVSI

- Prerequisite: Obtain d , a pilot sample of size n consisting of predicted risks and observed responses from the target population. 1. For j = 1 to M (number of Monte Carlo simulations): (a) Draw $D^{*}$ , a Bayesian bootstrap^†^ of the data d , as a random draw from the posterior distribution of the population that has generated the sample. This is done by assigning random weights^‡^ $W^{*}\sim \mathrm{Dirichlet}\left(1,1,...,1\right)$ to each observation in d . (b) Estimate $\theta^{*}$ , the true values of prevalence, sensitivity, and specificity in this iteration, from $D^{*}$ . (c) Calculate ${\mathrm{NB}}_{0}^{*}$ , ${\mathrm{NB}}_{1}^{*}$ , and ${\mathrm{NB}}_{2}^{*}$ , by plugging $\theta^{*}$ into equation 1. These are true NBs in this iteration. Record the maximum of true NBs: ${\mathrm{NB}}_{\mathrm{truth}}^{*}=\max\{{\mathrm{NB}}_{0}^{*},{\mathrm{NB}}_{1}^{*},{\mathrm{NB}}_{2}^{*}\}$ . (d) Draw D , a random realization of the data of the future study, through sampling N observations with replacement from $D^{*}$ . (e) Create $D^{+}=d+D$ , the pooled data of existing and future samples. (f) Calculate $\theta^{+}$ from $D^{+}$ , the updated estimates of prevalence, sensitivity, and specificity after obtaining the future sample. (g) Calculate ${\mathrm{NB}}_{0}^{+}$ , ${\mathrm{NB}}_{1}^{+}$ , and ${\mathrm{NB}}_{2}^{+}$ , updated estimates of expected NBs after obtaining the future sample, by plugging $\theta^{+}$ into equation 1. Determine the strategy that has the highest expected future NB: $l={\arg\;\max}_{i\in \{0,1,2\}}\left({\mathrm{NB}}_{i}^{+}\right)$ . Record the true NB of this strategy: ${\mathrm{NB}}_{\mathrm{sample}}^{*}={\mathrm{NB}}_{l}^{*}$ . - Next j . 2. Take the average of $NB^{*}$ s from step 1c and pick the maximum value. This is $\mathrm{EN}B_{\mathrm{current}}$ ^§^. 3. Average ${\mathrm{NB}}_{\mathrm{truth}}^{*}$ s from step 1c. This is $\mathrm{EN}B_{\mathrm{truth}}$ . From this subtract $\mathrm{EN}B_{\mathrm{current}}$ . This is the EVPI. 4. Average ${\mathrm{NB}}_{\mathrm{sample}}^{*}$ s from step 1g. This is $\mathrm{EN}B_{\mathrm{sample}}$ . From this subtract $\mathrm{EN}B_{\mathrm{current}}$ . This is the EVSI.

EVPI, expected value of perfect information; EVSI, expected value of sample information; ENB, expected net benefit; NB, net benefit.

† Ordinary bootstrap can also be used; see text.

‡ One way of generating such wights is by generating n exponential(1) random variables and scaling them to add up to 1. See the exemplary R code in Supplementary Material Section 1.

§ Because the NB terms are linear on the elements of θ , $\mathrm{EN}B_{\mathrm{current}}$ can also be directly evaluated in the original sample without bootstrapping, by plugging in the point estimates of θ into equation 1. However, we recommend averaging over bootstrapped values as the positive correlation between this term and the other term in EVPI and EVSI equations improves precision and prevents getting occasional negative value-of-information estimates.

The power of this approach is in the flexibility of the bootstrap method in accommodating different types of outcomes and practical considerations in validation studies. For example, if d has a nontrivial level of missing data, which affects the amount of information it contains, step 1c in Table 1 can include an imputation component, generating the population $D^{*}$ without any missing values (and similarly, step 1d can introduce missingness in the future sample to simulate a real-world validation dataset).

The ordinary bootstrap, which assigns weights from a scaled multinomial (1,1,…,1). distribution to observations in d , is conceptually similar to the Bayesian bootstrap and has been interpreted in a Bayesian way (e.g., in the approximate Bayesian bootstrap employed in missing value imputation^27^). In data-driven VoI analyses, it generates similar results to the Bayesian bootstrap.^25^ It can thus be used in lieu of the Bayesian bootstrap in step 1a.

### A Fast Algorithm Based on Beta-Binomial Modeling for Binary Outcomes

In their proposal for a parametric Bayesian NB estimation for binary outcomes, Netto Flores Cruz and Korthauer^28^ note that the likelihood function for NB can be factorized into terms involving prevalence, sensitivity, and specificity. As such, they take advantage of the beta-binomial conjugacy and note that specifying prior information in terms of independent beta distributions on these quantities will result in corresponding posterior distributions that are also independent beta. In this case, given that P(θ|d) is composed of 3 independent beta distributions, by the same token P(θ|d,D) after a realization of D will also be composed of 3 independent beta distributions. This results in a fast Monte Carlo approach for EVSI computation as explained in Table 2.

**Table 2:** Computation of EVSI Based on Beta-Binomial Modeling

- Prerequisite: Specify current information P(θ|d) as $\theta_{p}\sim \mathrm{Beta}\left(\alpha_{p},\beta_{p}\right)$ , $\theta_{\mathrm{se}}\sim \mathrm{Beta}\left(\alpha_{\mathrm{se}},\beta_{\mathrm{se}}\right)$ , $\theta_{\mathrm{sp}}\sim \mathrm{Beta}\left(\alpha_{\mathrm{sp}},\beta_{\mathrm{sp}}\right)$ . 1. For j = 1 to M (number of Monte Carlo simulations): (a) Obtain $\theta^{*}$ , by random drawing from P(θ|d) . These are true θ s in this iteration. (b) Calculate ${\mathrm{NB}}_{0}^{*}$ , ${\mathrm{NB}}_{1}^{*}$ , and ${\mathrm{NB}}_{2}^{*}$ , by plugging $\theta^{*}$ into equation 1. These are true NBs in this iteration. Record the maximum true NB: ${\mathrm{NB}}_{\mathrm{truth}}^{*}=\max\{{\mathrm{NB}}_{0}^{*},{\mathrm{NB}}_{1}^{*},{\mathrm{NB}}_{2}^{*}\}$ . (c) Generate D , the sample of future study, defined by $\left\{N_{\mathrm{tp}},N_{\mathrm{fn}},N_{\mathrm{tn}},N_{\mathrm{fp}}\right\}$ given $\theta^{*}$ obtained in step 1a as: $N_{+}\sim \mathrm{Binomial}\left(N,\theta_{p}^{*}\right)$ (number of positive cases in the future sample), $N_{\mathrm{tp}}\sim \mathrm{Binomial}\left(N_{+},\theta_{\mathrm{se}}^{*}\right)$ , $N_{\mathrm{fn}}=N_{+}-N_{\mathrm{tp}}$ , $N_{\mathrm{tn}}\sim \mathrm{Binomial}\left(N-N_{+},\theta_{\mathrm{sp}}^{*}\right)$ , $N_{\mathrm{fp}}=N-N_{+}-N_{\mathrm{tn}}$ . (d) Calculate $\theta^{+}$ , the updated estimates of prevalence, sensitivity, and specificity after obtaining the future sample: $\theta_{p}^{+}=\left(\alpha_{p}+N_{\mathrm{tp}}+N_{\mathrm{fn}}\right)/\left(\alpha_{p}+\beta_{p}+N\right)$ , $\theta_{\mathrm{se}}^{+}=\left(\alpha_{\mathrm{se}}+N_{\mathrm{tp}}\right)/\left(\alpha_{\mathrm{se}}+\beta_{\mathrm{se}}+N_{\mathrm{tp}}+N_{\mathrm{fn}}\right)$ , $\theta_{\mathrm{sp}}^{+}=\left(\alpha_{\mathrm{sp}}+N_{\mathrm{tn}}\right)/\left(\alpha_{\mathrm{sp}}+\beta_{\mathrm{sp}}+N_{\mathrm{tn}}+N_{\mathrm{fp}}\right)$ . (e) Calculate ${\mathrm{NB}}_{0}^{+}$ , ${\mathrm{NB}}_{1}^{+}$ , and ${\mathrm{NB}}_{2}^{+}$ , updated estimates of NBs after obtaining the future sample, by plugging $\theta^{+}$ into equation 1. Determine the strategy that has the highest expected future NB: $l={\arg\;\max}_{i\in \{0,1,2\}}\left({\mathrm{NB}}_{i}^{+}\right)$ . Record the true NB of this strategy: ${\mathrm{NB}}_{\mathrm{sample}}^{*}={\mathrm{NB}}_{l}^{*}$ . - Next j . 2. Take the average of $NB^{*}$ s from step 1b and pick the maximum value. This is $\mathrm{EN}B_{\mathrm{current}}$ . 3. Average ${\mathrm{NB}}_{\mathrm{truth}}^{*}$ s from step 1b. This is $\mathrm{EN}B_{\mathrm{truth}}$ . From this subtract $\mathrm{EN}B_{\mathrm{current}}$ . This is EVPI. 4. Average ${\mathrm{NB}}_{\mathrm{sample}}^{*}$ s from step 1e. This is $\mathrm{EN}B_{\mathrm{sample}}$ . From this subtract $\mathrm{EN}B_{\mathrm{current}}$ . This is EVSI.

EVPI, expected value of perfect information; EVSI, expected value of sample information; ENB, expected net benefit; NB, net benefit.

Independent beta distributions for each component of θ can emerge, for example, if we ask an expert to express their belief about prevalence, sensitivity, and specificity, in terms of fractions. That is, if their best estimate of prevalence is 30%, and their uncertainty is akin to their opinion coming from a sample of 10 individuals, the information can be expressed as $\theta_{p}\sim \mathrm{Beta}\left(3,7\right)$ (similar for sensitivity and specificity).

Importantly, for binary outcomes and in the absence of missing data, the Bayesian bootstrap approach explained previously reduces to this beta-binomial model. This is because the information in a sample d for estimating NBs can be fully specified via 4 quantities: the number of true positives ( $n_{\mathrm{tp}}$ ), false negatives ( $n_{\mathrm{fn}}$ ), true negatives ( $n_{\mathrm{tn}}$ ), and false positives ( $n_{\mathrm{fp}})$ . Due to the aggregate property of the Dirichlet distribution,^29^ the Dirichlet(0,0,...,0) prior used in the Bayesian bootstrap is equal to assigning Beta(0,0) priors to $\theta_{p}$ , $\theta_{\mathrm{se}}$ , and $\theta_{\mathrm{sp}}$ , resulting in the posterior distribution for the population that can be specified as $\left(p_{\mathrm{tp}},p_{\mathrm{fn}},p_{\mathrm{tn}},p_{\mathrm{fp}}\right)\sim \mathrm{Dirichlet}\left(n_{\mathrm{tp}},n_{\mathrm{fn}},n_{\mathrm{tn}},n_{\mathrm{fp}}\right)$ . As such, the beta-binomial algorithm will be equal to the Bayesian bootstrap and can be used instead for VoI computations due to its computational efficiency.

Yet another utility of this approach is that instead of the improper prior employed in the Bayesian bootstrap, the user can specify informative priors. Imagine in the current sample d of size n , there are m individuals who have experienced the outcome. If an independent cross-sectional study in the same target population has estimated outcome prevalence that can be expressed as Beta(α,β) , our current information about prevalence can be expressed as $\theta_{p}\sim \mathrm{Beta}\left(\alpha +m,\beta +n-m\right)$ . Even in the absence of external information, weakly informative priors can be employed to rectify some of the previously identified problems with bootstrap-based VoI calculations at extreme situations. In small validation samples and extreme thresholds, VoI calculations might generate counterintuitive results.^15^ One instance is when sample estimates of $\theta_{\mathrm{se}}$ or $\theta_{\mathrm{sp}}$ are at their extreme (0 or 1). The improper Beta(0,0) prior on these quantities results in improper beta posteriors with 1 of their 2 parameters being 0. This can be resolved if one uses a flat Beta(1,1) prior for these quantities, as noted by Netto Flores Cruz et al^28^.

### A General Algorithm for Arbitrary Specification of P(θ|d)

In many practical situations, our current information P(θ|d) may not have a simple mathematical form, but one can still draw a Monte Carlo sample of arbitrary size from its distribution. Examples include when $P\left(\theta_{p}\right)$ is constructed from a meta-analysis of prevalence studies, or $P\left(\theta_{\mathrm{se}},\theta_{\mathrm{sp}}\right)$ from a bivariate meta-analysis of the diagnostic accuracy of the test, giving rise to a joint distribution for $P\left(\theta_{\mathrm{se}},\theta_{\mathrm{sp}}\right)$ .^30^ Markov chain Monte Carlo methods can be used to obtain samples from the joint distribution of θ , even though the mathematical form of the joint density might be intractable (an example is the case studies in Wynants et al.^21^).

For such a general case in which we have a sample from P(θ|d) , the empirical distribution of the sample can be used as the distribution for the current information. We note that P(θ|d,D)∝P(θ)P(d,D|θ)=P(θ)P(d|θ)P(D|θ)∝P(θ|d)P(D|θ) . As such, the posterior distribution of θ after observing the future sample can be approximated by the discrete distribution that puts a mass $w^{\left(i\right)}\propto P\left(D|\theta^{\left(i\right)}\right)$ on the i th observation of the sample from P(θ|d) . This will result in the general algorithm explained in Table 3.

**Table 3:** EVSI Computation for Binary Outcomes for General, Nonconjugate Distributions

- Prerequisite: Specify current information as arbitrary distribution P(θ|d) . Obtain M draws $\left(\theta^{\left(1\right)},\theta^{\left(2\right)},...,\theta^{\left(M\right)}\right)$ from P(θ|d) . 1. For j = 1 to M (number of Monte Carlo simulations): (a) Let $\theta^{*}=\theta^{\left(i\right)}$ . This is true θ in this iteration.^†^ (b) Calculate ${\mathrm{NB}}_{0}^{*}$ , ${\mathrm{NB}}_{1}^{*}$ , and ${\mathrm{NB}}_{2}^{*}$ , by plugging $\theta^{*}$ into equation 1. These are true NBs in this iteration. Record the maximum true NB: ${\mathrm{NB}}_{\mathrm{truth}}^{*}=\max\{{\mathrm{NB}}_{0}^{*},{\mathrm{NB}}_{1}^{*},{\mathrm{NB}}_{2}^{*}\}$ . (c) Generate D , the sample of future study, defined by $\left\{N_{\mathrm{tp}},N_{\mathrm{fn}},N_{\mathrm{tn}},N_{\mathrm{fp}}\right\}$ as follows: $\;N_{+}\sim \mathrm{Binomial}\left(N,\theta_{p}^{*}\right)$ (number of positive cases in the future sample), $\;N_{\mathrm{tp}}\sim \mathrm{Binomial}\left(N_{+},\theta_{\mathrm{se}}^{*}\right)$ , $N_{\mathrm{fn}}=N_{+}-N_{\mathrm{tp}}$ , $\;N_{\mathrm{tn}}\sim \mathrm{Binomial}\left(N-N_{+},\theta_{\mathrm{sp}}^{*}\right)$ , $\;N_{\mathrm{fp}}=N-N_{+}-N_{\mathrm{tn}}$ . (d) Update the distribution of θ given the future sample. This is done by updating the weights $w^{\left(k\right)}$ based on the likelihood P(D|θ) . For binary outcomes the weights are: $w^{\left(k\right)}\propto P_{\mathrm{Bin}}\left(N_{\mathrm{tp}}+N_{\mathrm{fn}},N,\theta_{p}^{\left(k\right)}\right)\times P_{\mathrm{Bin}}\left(N_{\mathrm{tp}},N_{\mathrm{tp}}+N_{\mathrm{fn}},\theta_{\mathrm{se}}^{\left(k\right)}\right)\times P_{\mathrm{Bin}}\left(N_{\mathrm{tn}},N_{\mathrm{tn}}+N_{\mathrm{fp}},\theta_{\mathrm{sp}}^{\left(k\right)}\right)$ , with $P_{\mathrm{Bin}}\left(x,m,p\right)$ being the probability mass function of the binomial distribution at value x for m trials and success probability p . (e) Calculate ${\mathrm{NB}}_{0}^{+}$ , ${\mathrm{NB}}_{1}^{+}$ , and ${\mathrm{NB}}_{2}^{+}$ , updated estimates of expected NBs after obtaining the future sample, by using the weights generated in the previous step as follows: ${\mathrm{NB}}_{i}^{+}=\left\{ \begin{array}{cc} 0\;\;\;\;\;\;\;\;\;\;\;\;\;\;\;\;\;\;\;\;\;\;\;\;\;\;\;\;\;\;\;\;\;\;\;\;\;\;\;\;\;\;\;\;\;\;\;\;\;\;\;\;\;\;\;\;\;\;\;\;\;\;\;\;\;\;\;\;\;\;\;\;\;\;\;\;\;\;\;\;\;\;\;\;\;\; & i=0\\ \sum_{k=1}^{M}w^{\left(k\right)}\left\{\theta_{p}^{\left(k\right)}\theta_{\mathrm{se}}^{\left(k\right)}-\left(1-\theta_{p}^{\left(k\right)}\right)\left(1-\theta_{\mathrm{sp}}^{\left(k\right)}\right)\frac{z}{1-z}\right\}/\sum_{k=1}^{M}w^{\left(k\right)} & \;\;\;\;\;i=1\\ \sum_{k=1}^{M}w^{\left(k\right)}\left\{\theta_{p}^{\left(k\right)}-\left(1-\theta_{p}^{\left(k\right)}\right)\frac{z}{1-z}\right\}/\sum_{k=1}^{M}w^{\left(k\right)} & \;\;\;\;\;\;\;\;\;\;\;\;\;\;\;\;\;\;\;\;\;\;\;\;\;\;i=2 \end{array}\right..$ Determine the strategy that has the highest expected NB in this pooled sample: $l={\arg\;\max}_{i\in \{0,1,2\}}\left({\mathrm{NB}}_{i}^{+}\right)$ . Record the true NB of this strategy: ${\mathrm{NB}}_{\mathrm{sample}}^{*}={\mathrm{NB}}_{l}^{*}$ . - Next j . 2. Average ${\mathrm{NB}}_{\mathrm{truth}}^{*}$ s from from step 1b. This is $\mathrm{EN}B_{\mathrm{truth}}$ . Subtract from this $\mathrm{EN}B_{\mathrm{current}}$ . This is EVPI. 3. Average ${\mathrm{NB}}_{\mathrm{sample}}^{*}$ s from step 1e. This is $\mathrm{EN}B_{\mathrm{sample}}$ . Subtract from this $\mathrm{EN}B_{\mathrm{current}}$ . This is EVSI.

EVPI, expected value of perfect information; EVSI, expected value of sample information; ENB, expected net benefit; NB, net benefit.

† If the size of the sample is large, it might not be computationally feasible to loop over all observations. In which case one can pick one of $\theta^{\left(i\right)}s$ at random in this step.

### Case Study

We used data from GUSTO-I, a clinical trial of multiple thrombolytic strategies for acute myocardial infarction (AMI), as our case study.^31^ This dataset has been widely used for methodological research in predictive analytics.^32–34^ All analyses are conducted in R,^35^ with a fast implementation of the general EVSI algorithm in C++ (please refer to the *evsiexval* R package for details).

For the present case study, we use a similar setup as our previous work, with a risk prediction model for 30-day mortality developed using the non-US sample of this dataset and validated in the US subsample. The risk prediction model is a logistic regression model fitted to the entire non-US subset (sample size 17,796). We initially assume we have access to only n=500 individuals from the US subsample as the source of current information. We randomly selected 500 individuals, without replacement, from the entire 23,034 observations in the US subsample of GUSTO-I. Later, we will use more observations from the US subsample to study how EVSI behaves with the amount of current information. We consider the range of relevant thresholds to be 1% to 10%, with 1%, 2%, 5%, and 10% being of primary interest. VoI computations are based on 10^6^ Monte Carlo simulations using the aggregate beta-binomial model, with a flat Beta(1,1) prior on each component of θ .

Using this setup, we performed 2 simulation studies to explore the face validity of the proposed algorithms. In the first set, we aimed at comparing the numerical stability and computational time of the 3 algorithms. In the second set, we explored how EVSI changes as a function of the amount of current information, represented by n , the size of the current validation data from which P(θ|d) is constructed.

## Results

The prevalence of the outcome in the development sample (entire non-US sub-sample) was 0.0723; in the current validation sample it was 0.0860, while in the entire US sub-sample it was 0.0679. The risk prediction model was of the form:

$$\begin{array}{c} \mathrm{logit}\left(P\left(Y=1\right)\right)=-2.0842+0.0781\left[\mathrm{age}\right]+0.4027\\ \left[\mathrm{AMI}\;\mathrm{location}\;\mathrm{other}\;(\mathrm{vs}.\;\mathrm{inferior})\right]+0.5773\\ \left[\mathrm{AMI}\;\mathrm{location}\;\mathrm{anterior}(v.\;\mathrm{inferior})\right]+0.4678\\ \left[\mathrm{previous}\;\mathrm{AMI}\right]+0.7666\left[\mathrm{AMI}\;\mathrm{severity}(\mathrm{Killip}\;\mathrm{score})\right]\\ -0.0775\left[\min(\mathrm{blood}\;\mathrm{pressure},\;100)\right]+0.0182\left[\mathrm{pulse}\right]. \end{array}$$

**Figure 1:**
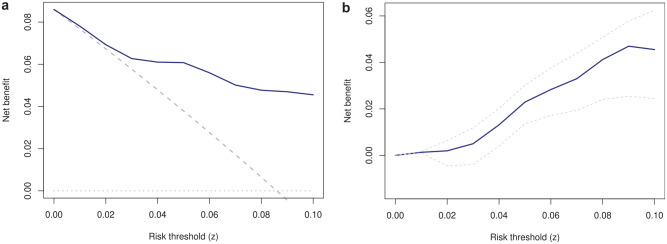
Decision curve (a) and incremental net benefit of the model versus the best alternative strategy (b). (a) Solid blue: use the model; dashed gray: treat all; dotted gray: treat none. (b) Dashed lines are 95% confidence intervals.

The decision curve depicting the NB of the model alongside alternative strategies is presented in Figure 1. Panel (a) shows the NB, and panel (b) shows the difference between the NB of the model and the best default strategy ( $NB_{1}-\max(NB_{0},NB_{2})$ ) along with its 95% CI (based on the percentile methods using 10,000 bootstraps). The model had higher ENB than the alternative strategies at the entire range of thresholds of interest.

Among 500 observations in the current sample, 43 experienced the outcome. Given the prior Beta(1,1) , this will result in $\theta_{p}\sim \mathrm{Beta}\left(44,458\right)$ . The distribution of sensitivity and specificity depends on the threshold. Taking z=0.02 as an example, given that 41 of the 43 individuals who experienced the outcome had a predicted risk ≥z , and 147 of the 457 who did not experience the outcome had predicted risk <z , with a Beta(1,1) prior, we have $\theta_{\mathrm{se}}\sim \mathrm{Beta}\left(42,3\right)$ and $\theta_{\mathrm{se}}\sim \mathrm{Beta}\left(148,311\right)$ .

**Figure 2:**
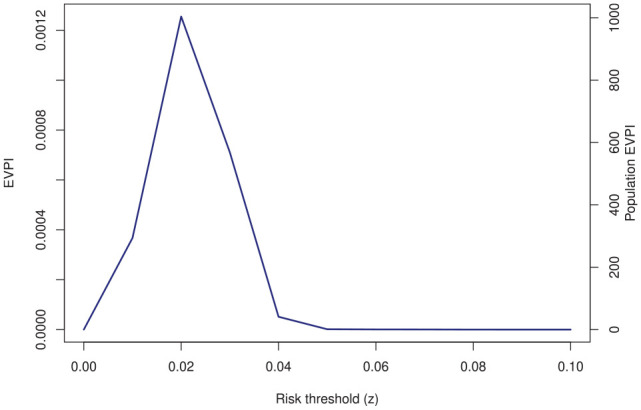
The expected value of perfect information (EVPI) of the validation sample.

The EVPI curve is presented in Figure 2. The EVPI at 0.01 threshold was 0.00037. At the 0.02 threshold it was 0.00125. The EVPI was 0 at 0.05 and 0.10 thresholds (so EVSI for any N will also be 0). We therefore focus on VoI calculations at 0.01 and 0.02 thresholds. EVPI quantifies the ENB loss per decision (every time the model might be used). The overall NB loss due to uncertainty is therefore affected by the expected number of times the medical decision will be made.^36^ In scaling VoI quantities to the population, we apply a similar approach as in our previous work^15^: there are about 800,000 AMIs in the United States every year,^37^ and a national guideline development panel in charge of recommending risk stratification for AMI management can consider all those instances relevant. The scaled VoI values are provided in true-positive units on the second *y*-axis of the EVPI figure. At the 0.02 threshold, a future validation study can have a maximum benefit equal to gaining 1,004 net true-positive cases (individuals who will die within 30 d and are correctly identified as high risk) per year, or avoiding 49,188 extra false-positive cases per year (individuals who will not die within 30 d but are identified as high risk).

**Figure 3:**
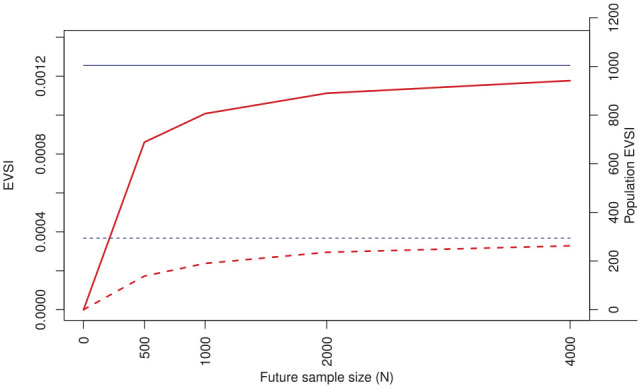
The expected value of sample information (EVSI) for the case study. Red: EVSI; blue (horizontal): the expected value of perfect information (EVPI). Dashed lines: z (risk threshold) = 0.01; solid lines: z = 0.02.

The EVSI curves are shown in Figure 3. Generally, it is expected that EVSI will increase with a larger future study and will asymptote to EVPI, a pattern that is obvious for both thresholds. The “diminishing return” pattern is also clear: the ENB gain steeply rises at small samples and plateaus as N grows, leaving little room for gaining NB beyond N = 4,000. Similar to EVPI, population scaling is applied to EVSIs and is presented as the second *y*-axis of the EVSI curve. At the 0.02 threshold, a future study of size N=1,000 has a per-decision EVSI value of 0.00101, corresponding to a population value of 806 in true-positive cases gained or 39,500 in false-positive cases averted.

Supplementary Material Section 2 provides results of the simulations comparing the 3 algorithms. All algorithms generated comparable VoI values and demonstrated the expected monotonicity behavior: the higher the N , the higher the EVSI, with EVSI asymptoting toward EVPI. The precision for the bootstrap-based and beta-binomial approaches was excellent with 10^6^ simulations (coefficient of variation [CV] of the Monte Carlo error <1%), with the latter being >30 times faster. On the other hand, the general algorithm had unacceptable variation (CV > 30%) when the size of the sample from P(θ|d) was 100, and the CV was still >10% with 1,000 samples, indicating that even larger samples might be required for reliable results. Supplementary Material Section 3 provides the results of brief simulation studies that show how the EVSI changes with the amount of current information. As expected, the higher the amount of current information (the larger the n ), the lower the gain in NB by conducting future studies. When current evidence is weak, the initial gain in NB by conducting future research can be very large. This is not the case when the current information is relatively robust (e.g., based on *n* = 8,000 observations, which includes on average 544 events).

## Discussion

Evaluating the performance of a model in a finite sample is fraught with uncertainty. In this work, we applied the VoI framework to investigate the decision-theoretic consequences of such uncertainty. We defined validation EVSI as the expected gain in NB by obtaining an external validation sample of a given size from the target population of interest. In a case study we showed the feasibility of EVSI calculations and studied how EVSI is affected by the amount of current information and the sample size of the future study. We suggested scaling the EVSI to the population to quantify the overall gain in clinical utility in true- (or false-) positive units from conducting an external validation study of a given sample size.

We proposed 3 algorithms for EVSI computations (with implementation in R as part of the *evsiexval* package: https://github.com/resplab/evsiexval). The bootstrap-based algorithm is applicable when previous individual-level data (e.g., from a pilot validation study) are at hand. The main advantage of this algorithm is in its flexibility, for example, in mimicking expected patterns of missingness in data. Among the 3 algorithms, this algorithm is the one that can most readily be extended to survival outcomes (similar to the extension of the bootstrap method for inference around NB^16^). However, this algorithm is applicable only when individual-level data are available and can be slow. The beta-binomial algorithm is applicable when current evidence on model performance can be expressed as independent beta distributions for prevalence, sensitivity, and specificity and is the fastest of the 3 algorithms. We showed that for binary outcomes and in the absence of censoring, this algorithm is equal to the Bayesian bootstrap (with the added flexibility that prior information can be incorporated). The general algorithm is the most versatile one as it works with any joint distribution of θ as long as one can obtain random draws from it. As such, this algorithm can be useful when P(θ|d) does not have a tractable form. However, the finite size of the sample adds another layer of uncertainty, potentially requiring significantly more computation time to achieve the same numerical accuracy of the other 2 algorithms.

How can EVSI analysis inform study design in predictive analytics? Conducting a validation study is an investment in resources that will generate further information on the performance of a clinical prediction model in a target population. The decision whether to undertake a validation study should ultimately hinge on whether the information gained from such a study is worth the required investments. The EVSI, when scaled to the population, determines the expected return on investment in NB unit. Ultimately, such return should be contrasted against the efforts and resources required for such a study. In decision-analytic (health policy) modeling, EVSI is typically in net monetary units, and when scaled to population, can be compared with the budget of a planned data collection activity. The optimal sample size will be one that maximizes the difference between population EVSI and study costs.^36^ The NB for risk prediction models, on the other hand, is in net true- or false-positive units and as such cannot directly be compared with the budget of a validation study. One can always embark on full decision analysis to translate all outcomes to net monetary units, but this will likely require sophisticated decision modeling and context-specific assumptions on long-term outcomes, a process that might take a significant amount of time and require further data collection (e.g., to obtain utility weights for outcomes). To us, a main reason for the vast popularity of decision curve analysis is that it provides an assumption-free, reproducible method for NB calculation based on the very same data that are used for studying model calibration and discrimination. We think VoI analysis during model development and validation should generally keep the same spirit. A full decision analysis should be relegated to after an impact analysis has measured the resource-use implications of implementing the model. This, however, means the decision rules for determining the optimal sample size of development and validation studies based on the VoI framework would be different than those used in health policy analysis. This article deliberately stayed away from proposing such rules and instead focused on defining concepts and proposing computation methods for EVSI. Proposing such decision rules is detached from EVSI calculations and deserves its own airing.

There are multiple areas of further inquiry. The EVSI framework should also be applied to the development phase of prediction models. This can guide the investigator on whether further development, or moving to validation, should be prioritized. We mainly focused on NB loss due to sampling uncertainty. However, there are several sources of uncertainty, such as whether our existing information on the performance of the model is directly applicable to the target population, or if predictors and outcome are measured with the same quality between the study and usual practice. The comparative statistical and computational performance of the EVSI computations algorithms, and the adequacy of a given number of simulations for each algorithm, should be evaluated in dedicated studies. We also do not claim the algorithms we proposed are the only ones that can be used for EVSI computations. Other algorithms, such as those based on the central limit theorem,^38,39^ can prove useful. VoI analysis in decision modeling has received a significant boost in computational speed in recent years due to the arrival of algorithms based on nonparametric regression modeling.^40,41^ This approach can facilitate VoI analysis in risk prediction as well. The EVSI defined in this work is for a single, homogeneous target population and does not consider heterogeneous settings. VoI metrics and corresponding computation algorithms for multicenter studies should be developed separately. Further, during external validation, often a secondary aim is to update the model if its performance turns out to be suboptimal.^42^ Such model revision can take different levels of complexity,^43^ and one might be interested in the expected yield of a given sample size for such model revision. This will get connected to the VoI concepts for model development. As stated earlier, how VoI metrics for prediction models should inform objective functions for determining the optimal sample size should be debated by the community in the hope of generating consensus and best practice standards.

There is an ongoing debate on the appropriateness of conventional metrics of uncertainty when reporting the NB of a clinical prediction model.^7–9^ The same concerns can logically be extended to the frequentist method for sample size and power calculations around NB.^5,6^ VoI methodology provides a rigorous, utilitarian response to such controversies. The toolbox of VoI methods for clinical prediction models is growing, and perhaps it is time to formalize the role of VoI in uncertainty quantification and design of empirical studies in predictive analytics.

## Supplemental Material

sj-docx-1-mdm-10.1177_0272989X251314010 – Supplemental material for Expected Value of Sample Information Calculations for Risk Prediction Model Validation Supplemental material, sj-docx-1-mdm-10.1177_0272989X251314010 for Expected Value of Sample Information Calculations for Risk Prediction Model Validation by Mohsen Sadatsafavi, Andrew J. Vickers, Tae Yoon Lee, Paul Gustafson and Laure Wynants in Medical Decision Making
