# Supplementary material for: Expected Value of Sample Information Calculations for Risk Prediction Model Validation
Source: Med Decis Making. 2025 Feb 18;45(3):232–44. doi: 10.1177/0272989X251314010 (PMC11894915; doi:10.1177/0272989X251314010)
Supplement: sj-docx-1-mdm-10.1177_0272989X251314010 – Supplemental material for Expected Value of Sample Information Calculations for Risk Prediction Model Validation [file sj-docx-1-mdm-10.1177_0272989X251314010.docx]

Supplementary Material for “Expected value of sample information calculations for risk prediction model validation”

Mohsen Sadatsafavi, Andrew J Vickers, Tae Yoon Lee, Paul Gustafson, Laure Wynants

November 21, 2024

Table of Contents

- Section 1: Exemplary R code for EVSI computation – Page 2
- Section 2: simulation study 1 – Page 9
- Section 3: simulation study 2 – Page 10
- Section 4: Code and data availability – Page 11

Section 1: Exemplary R code for EVSI computation

The purpose of this section is to demonstrate how the proposed algorithms for EVSI computations can be programmed in R.

The implementations here are for illustrative purposes and are not optimized for computational efficiency. The emphasis is to map the steps in the EVSI calculation algorithms to specific sections of the R code. Correspondingly, simulations are run with lower numbers than in the paper. For more efficient implementations please use the open-source *evsiexval* package <https://github.com/resplab/EVSIExVal>.

This tutorial is based on GUSTO-I data. EVSI is computed at 2% threshold and for a future study of N=1000.

*The code chunks below with default values should take in total 1-10 minutes to run on an average computer.

###

### **Data wrangling and general setup (required for all algorithms)**

We use data from the GUSTO-I trial. We fit a logistic regression model for predicting 30-day mortality in the non-US sample of the trial. We are interested in validating this model in the US sub-sample. We use a random subset of $n$=500 from the US sub-sample as the source of our ‘current’ information about the model’s NB, and calculate EVSIs for a future validation study of N=1000 based on these data.

set.seed(123)

library(predtools) #Contains the GUSTO data. Please install from CRAN or Github

z <- 0.02 #Risk threshold

N <- 1000 #Sample size of the future study

data(gusto)

gusto$kill <- (as.numeric(gusto$Killip)>1)*1

gusto$Y <- gusto$day30

data_us <- gusto[gusto$regl %in% c(1, 7, 9, 10, 11, 12, 14, 15),]

data_other <- gusto[!gusto$regl %in% c(1, 7, 9, 10, 11, 12, 14, 15),]

dev_data <- data_other

n <- 500 #Size of the current sample (d)

val_data <- data_us[sample(1:(dim(data_us)[1]),n,F),]

#This is the risk prediction model

model <- glm(Y~age+miloc+pmi+kill+pmin(sysbp,100)+pulse, data=dev_data,

             family=binomial(link="logit"))

#pi is the predicted risk

pi <- predict(model, type="response", newdata=val_data)

val_data$pi <- pi

### **Bootstrap-based algorithm**

For binary outcomes in the absence of censoring and missing predictors values, this algorithm will converge to the beta-binomial algorithm described later (which is much faster). This, we do not recommend this algorithm for binary response and complete data. The main utility of the algorithm would be in dealing with other types of outcomes or when there is non-ignorable amount of missingness.

Note that in this two-level resampling algorithm, we implement bootstrapping via assigning weights to the observations, instead of creating resampled datasets. These are equal but the latter algorithm can involve memory allocation which will slow down the process.

set.seed(1)

M <- 10^5 #Number of Monte Carlo simulations

#.s stands for * superscript and .p for + in corresponding algorithmic description

NB1.s <- NB2.s <- NBtruth.s <- NB1.p <- NB2.p <- NBsample.s <- rep(0,M)

#Main simulation loop

for(j in 1:M)

{

  #Bayesian bootstrapping involves sampling from Dirichlet(1,1,...,1).

  #Here we generate weights W by normalizing Gamma(1.1)=Exponential(1) random variables.

  ###Step 1a

  W <- rexp(n, 1)

  W <- W/sum(W)

  ###Step 1b (true values of prevalence, sensitivity, and specificity)

  prevs <- sum(val_data$Y*W)/sum(W)

  ses <- sum(val_data$Y*(val_data$pi>=z)*W)/sum(val_data$Y*W)

  sps <- sum((1-val_data$Y)*(val_data$pi<z)*W)/sum((1-val_data$Y)*W)

  ###Step 1c

  #These are draws from 'true' NBs (NB* - note that NB0s=0)

  NB1.s[j] <- prevs*ses-(1-prevs)*(1-sps)*z/(1-z)

  NB2.s[j] <- prevs-(1-prevs)*z/(1-z)

  NBtruth.s[j] <- max(0, NB1.s[j], NB2.s[j])

  ###Step 1d

  #Second-level resampling creates D, future dataset.

  #Note that resampling can be done via weighting by multinational distribution

  W_D <- rmultinom(1, N, W)

  #Imputing missing predictor values should be implemented at this point

  #(not relevant for GUSTO data)

  ###Step 1e: pooling the current and future samples

  #Because each observation in the original sample d has a weight of 1,

  #adding +1 to all weights represents pooling the current and future samples

  W_pooled <- W_D+1

  ###Step 1f: updating parameters after observing future data

  prev.p <- sum(val_data$Y*W_pooled)/sum(W_pooled)

  se.p <- sum(val_data$Y*(val_data$pi>=z)*W_pooled)/sum(val_data$Y*W_pooled)

  sp.p <- sum((1-val_data$Y)*(val_data$pi<z)*W_pooled)/sum((1-val_data$Y)*W_pooled)

  ###Step 1g: updating NBs after observing future data

  NB1.p[j] <- prev.p*se.p-(1-prev.p)*(1-sp.p)*z/(1-z)

  NB2.p[j] <- prev.p-(1-prev.p)*z/(1-z)

  l <- which.max(c(0,NB1.p[j],NB2.p[j])) #Winning strategy

  NBsample.s[j] <- c(0,NB1.s[j],NB2.s[j])[l] #True NB of the winning strategy

}

###Step 2

ENBcurrent <- max(0, mean(NB1.s), mean(NB2.s))

###Step 3

EVPI <- mean(NBtruth.s) - ENBcurrent

###Step 4

EVSI <- mean(NBsample.s) - ENBcurrent

print(EVPI)

## [1] 0.0005066706

print(EVSI)

## [1] 0.0003473902

### **Beta-binomial method**

For binary outcomes and in the absence of missing values, the bootstrapped-based algorithm can be done in aggregate due to the beta-binomial conjugacy. As stated in the main text, the sample can be summarized by its sample size as well as outcome prevalence, sensitivity, and specificity, with likelihood function from the data being a product of independent beta PDFs for prevalence, sensitivity, and specificity. Calculations can thus be performed in aggregate.

The implementation of this code in the *evsiexval* package is vectorized. Unvectorized R code is provided here because it provides a direct mapping with the steps provided in Table 2 of the main text.

NOTE: To keep the results consistent with the bootstrap method, we are assigning Beta(0,0) to all three parameters (which is the implied prior in the Bayesian bootstrap). Note that the results in the main text are based on Beta(1,1) priors, which is what we generally recommend for the reason explained in the main text.

set.seed(1)

n <- nrow(val_data)

nD <- sum(val_data$Y)

ntp <- sum(val_data$Y*(val_data$pi>=z))

nfn <- nD-ntp

ntn <- sum((1-val_data$Y)*(val_data$pi<z))

nfp <- n-nD-ntn

evidence <- list(prev=c(nD, n-nD),

                 se=c(ntp, nfn),

                 sp=c(ntn, nfp))

M <- 10^6 #Number of Monte Carlo simulations

#.s stands for * superscript and .p for + in the corresponding algorithmic description

NB1.s <- NB2.s <- NBtruth.s <- NBsample.s <- rep(0,M)

for(j in 1:M)

{

  ###Step 1a

  prevs <- rbeta(1, evidence$prev[1], evidence$prev[2])

  ses <- rbeta(1, evidence$se[1], evidence$se[2])

  sps <- rbeta(1, evidence$sp[1], evidence$sp[2])

  ###Step 1b

  NB1.s[j] <- prevs*ses-(1-prevs)*(1-sps)*z/(1-z)

  NB2.s[j] <- prevs-(1-prevs)*z/(1-z)

  NBtruth.s[j] <- max(0, NB1.s[j], NB2.s[j])

  ### Step 1c

  #Generating D which can be summarized in terms of true/false positive/negative frequencies

  Nplus <- rbinom(1, size=N, prob=prevs)

  Ntp <- rbinom(1, size=Nplus, prob=ses)

  Nfn <- Nplus-Ntp

  Ntn <- rbinom(1, size=N-Nplus, prob=sps)

  Nfp <- N-Nplus-Ntn

  ### Step 1d: Update parameters after observing the future sample

  prev.p <- (evidence$prev[1]+Ntp+Nfn)/(evidence$prev[1]+evidence$prev[2]+N)

  se.p <- (evidence$se[1]+Ntp)/(evidence$se[1]+evidence$se[2]+Ntp+Nfn)

  sp.p <- (evidence$sp[1]+Ntn)/(evidence$sp[1]+evidence$sp[2]+Ntn+Nfp)

  ### Step 1e: Update NBs after observing the future sample

  NB1.p <- prev.p*se.p-(1-prev.p)*(1-sp.p)*z/(1-z)

  NB2.p <- prev.p-(1-prev.p)*z/(1-z)

  l <- which.max(c(0,NB1.p,NB2.p)) #Winning strategy

  NBsample.s[j] <- c(0,NB1.s[j],NB2.s[j])[l] #True NB of the winning strategy

}

###Step 2

ENBcurrent <- max(0, mean(NB1.s), mean(NB1.s))

###Step 3

EVPI <- mean(NBtruth.s) - ENBcurrent

###Step 4

EVSI <- mean(NBsample.s) - ENBcurrent

print(EVPI)

## [1] 0.0005064143

print(EVSI)

## [1] 0.0003463335

### **General, sample-based method**

This algorithm is a general one that does not require the current information $P\left( \theta|d \right)$ to be in an expressible mathematical form. Instead, it requires a sample from the (joint) posterior distribution of (prev, se, sp). This is particularly relevant for situations where we are using MCMC methods to generate posterior samples from a complex model (e.g., a random-effects model for joint inference on sensitivity and specificity of the model at the threshold of interest).

This algorithm has a complexity of $O\left( M^{3} \right)$, where M is the size of the sample. This can quickly become overwhelming for R. The evsiexval package implements this algorithm in C++ (which is used for the results reported in the paper – this implementation also allows separating the inner and outer simulation sizes for more control).

The R code below is inevitably vectorized to avoid long computation times, and the size of the sample is kept at 250. This is not generally sufficient for a real analysis. Instead of an outer simulation that samples from each observation, we are looping over the 250 observations (in vectorized code). This removes the unnecessary Monte Carlo error for the outer simulation. However, this will not be practical if the sample from the posterior distribution is large.

set.seed(1)

M <- 250 #Number of Monte Carlo simulations

n <- nrow(val_data)

n_D <- sum(val_data$Y)

n_tp <- sum(val_data$Y*(val_data$pi>=z))

n_fn <- n_D-n_tp

n_tn <- sum((1-val_data$Y)*(val_data$pi<z))

n_fp <- n-n_D-n_tn

evidence <- list(prev=c(n_D, n-n_D),

                 se=c(n_tp, n_fn),

                 sp=c(n_tn, n_fp))

samples <- cbind(prev=rbeta(M, evidence$prev[1],evidence$prev[2]),

                 se=rbeta(M, evidence$se[1],evidence$se[2]),

                 sp=rbeta(M, evidence$sp[1],evidence$sp[2])

)

###Step 1a is not applicable as the code is vectorized

###Step 1b

#Vectorized calculation of NBs

NB.s <- cbind(0,

              samples[,1]*samples[,2]-(1-samples[,1])*(1-samples[,3])*z/(1-z),

              samples[,1]-(1-samples[,1])*z/(1-z)

)

NBtruth.s <- apply(NB.s, 1, max)

###Step 1c

#Replicating the sample M times such that we can vectorize the calculations

#WARNING: This part is RAM-intensive as it creates matrices with M*n_sim rows.

#If M is large and this will not be practical, create a new sample...

#that is a subset of the original sample before the below line.

S <- do.call(rbind, replicate(M, samples, simplify=FALSE))

#Data for the future study for every row of S

Nplus <- rbinom(nrow(S), size=N, prob=S[,1])

Ntp <- rbinom(nrow(S), size=Nplus, prob=S[,2])

Ntn <- rbinom(nrow(S), size=N-Nplus, prob=S[,3])

###Steps 1d and 1e

#Creating the weight vector outside the function to minimize memory reallocation

w <- double(nrow(samples))

NB_pooled <- matrix(double(1), nrow=nrow(samples),ncol=3)

#Takes one realization of future study, updates the evidence

#Returns the highest NB

find_winner <- function(X)

{

  #This is the likelihood

  ###1d: generate weights

  w <- dbinom(X[1], N, samples[,1])*

    dbinom(X[2], X[1], samples[,2])*

    dbinom(X[3], N-X[1], samples[,3])

  ###1e: NB**s and picking the maximum

  max(0,

      sum(w*(samples[,1]*samples[,2]-(1-samples[,1])*(1-samples[,3])*z/(1-z)))/sum(w),

      sum(w*(samples[,1]-(1-samples[,1])*z/(1-z))/sum(w))

  )

}

#Warning: this step might take some CPU time

NBsample.s <- apply(cbind(Nplus, Ntp, Ntn), 1, find_winner)

###Step 2

ENBcurrent <- max(colMeans(NB.s))

###Step 3

EVPI <- mean(NBtruth.s) - ENBcurrent

###Step 4

EVSI <- mean(NBsample.s) - ENBcurrent

print(EVPI)

## [1] 0.0005415935

print(EVSI)

## [1] 0.0003323871

Section 2: simulation study 1

We explored how EVSI changes as a function of the amount of current information, represented by n, the size of d, the current validation data from which P(θ|d) is constructed. Starting from a value of 500, we doubled n, specified the distributions for θp, θse, and θsp based on this sample, and performed EVSI calculations as above, until reaching n = 8,000. Results are provided for both 0.01 and 0.02 thresholds and are the average of 100 independent simulations, in each a new sample (with replacement) from the US subset of GUSTO-I was obtained as the current validation data (100 simulations were considered adequate based on inspecting the standard error of the Monte-Carlo simulations). Results are presented in ***Figure S1***.

***Figure S1:*** Average expected value of sample information (EVSI) as a function of the sample size of the future study (*D^∗^*) for various levels of current information, represented by the sample size of the current study (*n*) from which *P*(θ) is constructed (results are average of 100 simulations). Black: *n* = 500; blue: *n* = 1*,*000; green: *n* = 2*,*000; orange: *n* = 4*,*000; red: *n* = 8*,*000. Dashed lines: *z* (risk threshold)=0.01; solid lines: *z*=0.02


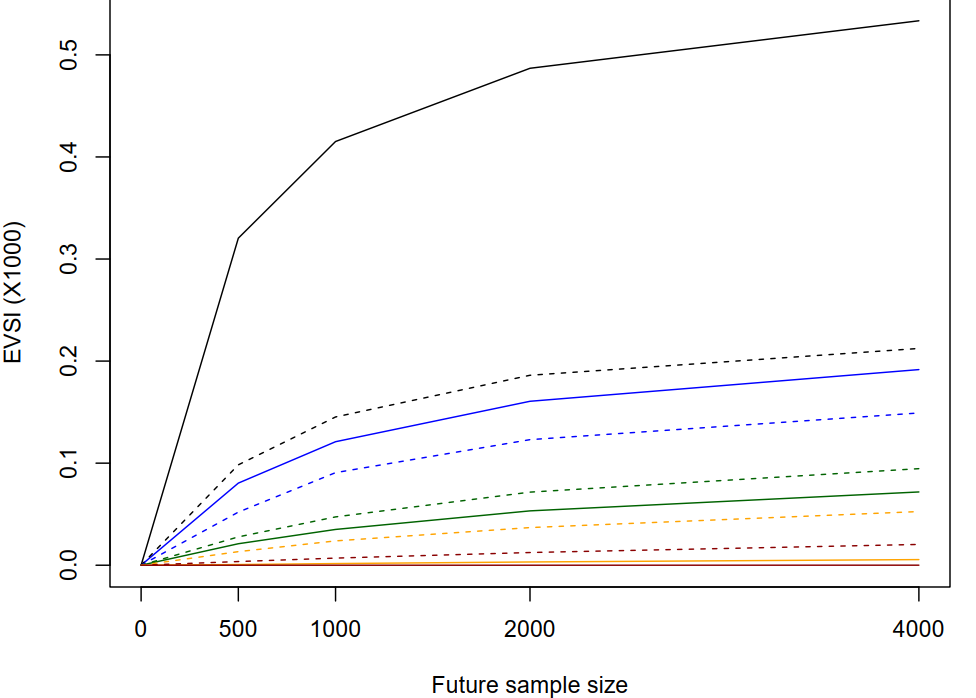


Section 3: simulation study 2

The second simulation study was aimed at comparing the numerical stability and computational time of the three algorithms. We reported the mean and coefficient of variation (CV, defined as standard error divided by the mean) of EVSI and EVPI values across 100 independent simulations for each algorithm. Each run was based on 10^6 internal simulations. The general algorithm has an extra free parameter: the size of the sample (M). We therefore repeated the simulations for this algorithm using M=100 and M=1,000. As the Bayesian bootstrap method implies a Beta(0,0) prior on θ, to make the results comparable, we used the same prior for the other algorithms, as opposed to the Beta(1,1) used in the case study. Computations are performed using single-threaded code in R version 4.4.0 on a Windows PC with an Intel® Core™ i9-9900T CPU @ 2.10GHz with 32GB RAM. Results are presented in ***Table S1***.

***Table S1.*** Results of the simulation studies (z=0.02). Values are average over 100 independent simulations (values in bracket are the coefficient of variation [CV], defined as the standard deviation of VoI quantities divided by their mean).

|  | **EVSI (N=500)** | **EVSI (N=1,000)** | **EVSI (N=2,000)** | **EVSI (N=4,000)** | **EVPI** | **CPU time (seconds)*** |
| --- | --- | --- | --- | --- | --- | --- |
| Bayesian bootstrap | 2.604e-04 (5.18e-03) | 3.473e-04 (4.01e-03) | 4.131e-04 (3.40e-03) | 4.555e-04 (3.07e-03) | 5.069e-04 (2.74e-03) | 446.8 |
| Regular (approximate Bayesian) bootstrap | 2.162e-04 (5.11e-03) | 3.004e-04 (3.97e-03) | 3.696e-04 (3.43e-03) | 4.173e-04 (2.99e-03) | 4.692e-04 (2.71e-03) | 392.5 |
| Beta-binomial | 2.607e-04 (5.22e-03) | 3.476e-04 (3.83e-03) | 4.135e-04 (3.32e-03) | 4.558e-04 (2.94e-03) | 5.073e-04 (2.59e-03) | 12.2 |
| General  (M=100) | 2.496e-04 (4.53e-01) | 3.391e-04 (3.81e-01) | 4.067e-04 (3.37e-01) | 4.493e-04 (3.13e-01) | 4.896e-04 (2.89e-01) | 16 |
| General  (M=1000) | 2.546e-04 (1.47e-01) | 3.454e-04 (1.25e-01) | 4.134e-04 (1.09e-01) | 4.567e-04 (1.00e-01) | 5.063e-04 (9.06e-02) | 148.9 |

*EVSI: expected value of sample information; EVPI: expected value of perfect information; CPU: central processing unit*

*For every algorithm, some components of computation are common across all N values (e.g., the first level of bootstrapping in the bootstrap-based methods). As such, EVSI calculations are done across a range of N values.

Section 4: Code and data availability

All the code and data that are used to generate the results in this paper are provided to reviewers and will be made publicly available shall the manuscript is accepted for publication.

• The GUSTO data are available from the *predtools* package (from CRAN or GitHub: <https://github.com/resplab/predtools>)

• The core functions are accessible by building the *evsiexval* R package (in the R folder), available from <https://github.com/resplab/EVSIExVal>

• The analysis code is provided here: <https://github.com/resplab/papercode/tree/main/evsiexval>

All the results are reproducible by the code in the analysis folder

- The case study can be reproduced by running case_study.R
- The simulation study 1 results can be reproduced by running gusto_sim.R
- The simulation study 2 results can be reproduced by running gusto_evsi_compare.R

NOTE: The code requires the *evsiexval* package to have been compiled and installed. It also requires sourcing the include.R file. Please ensure the path to this file on your computer is correct.

• The Supplementary Material contains a stand-alone markdown file that illustrate the implementation of the algorithms without requiring access to the GitHub repo or package compilation
